# The Geomagnetic Field Is a Contributing Factor for an Efficient Iron Uptake in *Arabidopsis thaliana*

**DOI:** 10.3389/fpls.2020.00325

**Published:** 2020-04-21

**Authors:** Monirul Islam, Massimo E. Maffei, Gianpiero Vigani

**Affiliations:** Department of Life Sciences and Systems Biology, Innovation Centre, University of Turin, Turin, Italy

**Keywords:** Fe deficiency, iron uptake, magnetic field, root metal homeostasis, near-null magnetic field

## Abstract

The Earth’s magnetic field, defined as the geomagnetic field (GMF), is an unavoidable environmental factor for all living organisms. Variation in the GMF intensity was found to affect the content of some nutrients and their associated channels and transporters in *Arabidopsis thaliana*. In this work, we observed that reduction of the GMF to near null magnetic field (NNMF) affects the accumulation of metals in plant tissues, mainly iron (Fe) and zinc (Zn) content, while the content of others metals such as copper (Cu) and manganese (Mn) is not affected. Accordingly, Fe uptake genes were induced in the roots of NNMF-exposed plants and the root Fe reductase activity was affected by transferring GMF-exposed plant to NNMF condition. Under Fe deficiency, NNMF-exposed plants displayed a limitation in the activation of Fe-deficiency induced genes. Such an effect was associated with the strong accumulation of Zn and Cu observed under NNMF conditions. Overall, our results provide evidence on the important role of the GMF on the iron uptake efficiency of plants.

## Introduction

Plant growth depends on a variety of environmental factors, including light, temperature, and the availability of both water and nutrients. Impairment of such factors strongly influences plant growth and morphogenesis (Bechtold and Field, 2018). However, other abiotic factors such as electric, radioactive, seismic, georthermal, gravitational, geochemical, and magnetic fields (MF) represent both important stress factors and unavoidable conditions during plant growth (Belyavskaya, 2004; Nyakane et al., 2019). Living organisms, including plants, can perceive a variation in one or more of these force fields (Nyakane et al., 2019). Indeed, plants perceive light and gravity variations by activating the so-called phototropic and gravitropic responses, respectively (Hoson, 2014; Liscum et al., 2014).

In both animal and plants, the mechanism of perception of the Earth’s magnetic field, also known the geomagnetic field (GMF), is still not fully understood. GMF is an unavoidable environmental factor for all living organisms, and it influences many biological processes. Over the past years, research carried out on the effect of magnetic fields on plants have been thoroughly reviewed (Phirke et al., 1996; Abe et al., 1997; Belyavskaya, 2004; Minorsky, 2007; Teixeira da Silva and Dobránszki, 2015, 2016) and indicates the presence of a still uncharacterized plant magnetoreceptor. In plants, recent reports revealed that variation of MF intensity affects photoreceptor activity (Agliassa et al., 2018b) and light-dependent processes such as leaf movement, stomatal conductance, and chlorophyll content (Galland and Pazur, 2005; Maffei, 2014). Furthermore, changes in the hormone levels, for instance, auxin and gibberellin, as well as a delay in flowering time have also been observed under near null MF (NNMF) conditions (Xu et al., 2012, 2017, 2018; Agliassa et al., 2018a). Such delayed transition to flowering under NNMF is likely involved in the Angiosperms speciation during GMF reversals (Maffei, 2014; Occhipinti et al., 2014; Bertea et al., 2015), suggesting a possible contribution of the GMF magnitude on plant evolution.

Recently, the reduction of the GMF to NNMF was found to affect the nutrient status of plants. Indeed, the content of essential inorganic cations (such as Ca, K, Mg) and anions (such as NO_3_^–^, SO_4_^–^, PO_4_^2=^) and the gene expression of their transporters and channels are affected under NNMF conditions (Narayana et al., 2018). Other than such ions, additional elements such as iron (Fe), copper (Cu), zinc (Zn), manganese (Mn), are essential for plant growth (Palmer and Guerinot, 2009; Marschner, 2011). Iron is required for photosynthetic and respiratory electron transport chains, DNA synthesis, nitrogen fixation, hormone synthesis (Vigani and Murgia, 2018). Manganese is essential for the oxygen-evolving complex and for other enzymes like Mn-superoxide dismutase (Marschner, 2011). Like Fe, also Cu is an essential cofactor of electron-transfer reactions mediated by proteins involved in photosynthesis and respiration (plastocyanin and cytochrome oxidase) (Clemens, 2001; Peñarrubia et al., 2010; Vigani and Hanikenne, 2018). Zinc is a structural constituent of many proteins as well as a cofactor for some enzymes (e.g., metalloproteinases, Cu-Zn superoxide dismutase, and carbonic anhydrase) (Krämer and Clemens, 2006; Escudero-Almanza et al., 2012; Sinclair and Kramer, 2012). So far, no clear information is available about the effect of MF variation on metals homeostasis in plants and this work aims to provide new insight in such topic.

Deciphering the effect of geophysical fields, such as the GMF, on plants is challenging because specific experimental conditions are difficult to be realized. For example, in the case of gravitational force, studies have been conducted establishing microgravity condition by using a rotator clinostat or by performing experiment directly in microgravity conditions (Hoson, 2014 and references therein). One way to demonstrate the role of the GMF on plants is to expose plants in conditions where the GMF is altered, by either increasing or reducing the MF flux. In space programs, humans, animals and plants are introduced into magnetic environments with a decrease of MF intensity by about three orders of magnitude respect to GMF intensity (i.e., from about 35 μT to near 1 nT) (Maffei, 2014). Such condition is achievable by using GMF compensation systems such as the one we developed by using a triaxial Helmholtz coil (three computer-controlled orthogonal Helmholtz coils) (Bertea et al., 2015; Agliassa et al., 2018a). Our strategy to assess the role of the GMF on metal uptake in Arabidopsis seedlings was through the comparison of plants grown under either GMF or NNMF conditions. Here, we show that NNMF-exposed plants displayed Fe deficiency-induced responses along with an impairment of root Fe reductase activity, thus indicating the critical role of the natural GMF on metal uptake.

## Materials and Methods

### Plant Materials, Media Composition and Growth Conditions

Seeds of *Arabidopsis thaliana* ecotype Columbia-0 (Col-0) wild type (WT) and *spl7* mutant kindly provided by Dr. Maria Bernal and Prof. Ute Krämer and previously characterized in Bernal et al. (2012), were surface sterilized with 70% v/v ethanol for 2 min and then with 5% w/v calcium hypochlorite for 5 min. After 3–5 washes with sterile water, seeds were sown on the surface of sterile agar plates (12 × 12 cm) with the medium composition according to Gruber et al. (2013) (Supplementary Table S1). Plants were grown in the presence (+Fe) or in the absence (−Fe) of Fe(III)EDTA. Furthermore, 300 μM ferroZine (−FeFRZ) was added to the –Fe media to further decrease Fe availability condition. Plates were sealed with Micropore tape to allow gas exchange and to avoid condensation. Plates were then vernalized for 48 h and then exposed vertically under a homogenous and continuous light source at 120 μmolm^–2^ s^–1^ and 22°C (±1.5) for 14 h to induce germination and to maintain the correct geotropic position of roots and shoots before being kept in the darkness at room temperature for 72 h. Plates were then transferred, in the same laboratory and at the same time, under either NNMF or GMF (controls) and exposed to 130 μmol m^–2^ s^–1^ white light provided by a high-pressure sodium lamp source (SILVANIA, Grolux 600W, Belgium) by using a light blue spotlight film to reduce the red component of lamps at 22°C (±1.5°C) with a 16/8 light/darkness photoperiod. All experiments were performed under normal gravity.

### GMF Reduction System and Plant Exposure

The GMF (or local GMF) values were typical of the Northern hemisphere at 45°0′5″ N and 7°36′58″ E coordinates ranging between 41 and 43 μT. NNMF was generated by three orthogonal Helmholtz coils as previously detailed (Agliassa et al., 2018a) to values ranging between 40 to 44 nT. Because GMF is the natural condition experienced by plants, we chose to use GMF as control. According to a previous characterization performed in our lab (Narayana et al., 2018), Arabidopsis seedlings containing plates were then exposed for 1 h, 4 h, 24 h, 48 h, and 96 h to either NNMF or GMF (controls).

### Metals Content Determination

Shoot and root samples collected from plants (wt and *spl7* lines) grown under +Fe and –Fe conditions and exposed to either GMF or NNMF for 96 h were dried in a ventilated oven (70°C) for 4 days. Dry weight was measured. Sampled tissues were then digested by using 65% HNO_3_ at 120°C. The mineralized samples were transferred into polypropylene test tubes. Samples were diluted 1:40 in MILLI-Q water and the concentration of metals elements was measured by Inductively Coupled Plasma-Mass Spectrometry ICP-MS (BRUKER Aurora-M90 ICP-MS) as previously described by Vigani et al. (2017) and Barocchi et al. (2018).

### Root Cu (II) Reductase and Fe (III) Chelate Reductase Activities

Cu (II) and Fe (III) chelate reductases activities were determined on the root surface by use of 7 days-old intact roots seedlings grown in sterile plates exposed to either GMF or NNMF. The assays were performed according to Bernal et al. (2012). The assay solution consisted of 0.1 mM Fe (III) NaEDTA and 0.3 mM FerroZine [3-(2-pyridyl)-5,6-diphenyl-1,2,4-triazine-4′,4″-disulfonic acid] (Sigma-Aldrich) in ultrapure water for measurement of Fe(III) chelate reductase activity or 0.2 mM CuSO_4_, 0.6 mM Na_3_-citrate, and 0.4 mM Bathocuproinedisulfonic acid disodium salt hydrate [(BCDS) Sigma-Aldrich] in ultrapure water for measurement of Cu (II) reductase activity, respectively. The assays were conducted on pooled roots (from 20 seedlings) from (i) GMF-exposed plants and NNMF-exposed plants in the dark and at room temperature and (ii) after transferring GMF-exposed plants to NNMF and *viceversa*. After 30 min for Fe(III) chelate reductase and 20 min for Cu (II) reductase, the absorbance of assay solutions was measured at 562 nm for the Fe(II)-FerroZine complex and 483 nm for the Cu(I)-BCDS complex, respectively. During the exposure time of plants roots in solution, Eppendorf tubes were slightly shaken to allow roots to increase their reductase activity. Extinction coefficients were 28.6 mM^–1^ cm^–1^ for the Fe (II) FerroZine complex (Gibbs, 1976) and 12.25 mM^–1^ cm^–1^ for the Cu (I) BCDS complex (Welch et al., 1993).

### RNA Purification and cDNA Synthesis From Arabidopsis Roots

*Arabidopsis thaliana* WT plants roots and shoots were separately collected for each time point (1 h, 4 h, 24 h, 48 h, and 96 h) from either GMF or NNMF grown plants and immediately frozen in liquid nitrogen. Samples were kept at −80°C for further analysis. For each time point, 20 mg frozen plant material was ground in liquid nitrogen with a mortar and pestle. Total RNA was isolated using the Machery-Nagel RNA Isolation mini Kit (Machery-Nagel GmbH & Co., Düren, Germany), and RNase-Free DNase, in accordance with the manufacturer’s protocols. RNA quality and quantity was checked spectrophotometrically (Pharmacia Biotech Ultrospec 3000, United States). cDNA was synthesized starting from 1 μg RNA using the High Capacity cDNA Reverse Transcription kit (Applied Biosystem, Foster City, CA, United States), in accordance with the manufacturer’s recommendations.

### Quantitative Real Time-PCR (qPCR)

Quantitative real time-PCR was performed on cDNA obtained from roots of plants harvested 1 h, 4 h, 24 h, 48 h, and 96 h after exposure to GMF and NNMF conditions. qPCR experiments were run on an Mx3000P Real-Time System (Stratagene, La Jolla, CA, United States) using SYBR green I with ROX as an internal loading standard. The reaction mixture was 10 μl and comprised of 5 μl of 2 × Maxima SYBR Green qPCR Master Mix (Thermo Fisher Scientific), 0.5 μl of cDNA, and 100 nM primers (Integrated DNA Technologies, Coralville, IA, United States). Non-template controls were also included. Primers were designed using Primer 3.0 software (Rozen and Skaletsky, 2000) and the relative sequences are listed in Supplementary Table S2. Controls included non-RT controls (using total RNA without reverse transcription to monitor for genomic DNA contamination) and non-template controls (water template). Specifically, PCR conditions were: 10 min at 95°C, 45 cycles of 10 s at 95°C, 15 s at 55°C, and 20 s at 72°C and all runs were followed by a melting curve analysis with the following gradient: 1 min at 95°C, 15 s at 55°C, 10 s at 95°C for the genes *At4g30190*, PLASMA MEMBRANE PROTON ATPASE 2 (*AHA2*); *At1g01580*, FERRIC REDUCTION OXIDASE 2; *At4g19690*, IRON-REGULATED TRANSPORTER 1, *IRT1*; *At2g28160*, FER-LIKE IRON DEFICIENCY INDUCED TRANSCRIPTION FACTOR, *FIT*; *At3g56970*, BASIC HELIX-LOOP-HELIX 38; *At3g56980*, BASIC HELIX-LOOP-HELIX 39; *At5g54680*, BASIC HELIX-LOOP-HELIX 105 (*ILR3*).

Four different reference genes (At2g37620, ACTIN1 (ACT1); At5g19510, ELONGATION FACTOR 1B ALPHA-SUBUNIT 2 (eEF1Balpha2); At1g13440, CYTOPLASMIC GLYCERALDEHYDE-3-PHOSPHATE DEHYDROGENASE (GAPC2); and At1g51710, UBIQUITIN SPECIFIC PROTEASE 6 (UBP6) were used to normalize the qPCR results from NNMF and GMF samples. As previously described in Agliassa et al. (2018a) the most stable gene identified by using Normfinder software (MOMA, Aarhus, Denmark) (Andersen et al., 2004) was eEF1Balpha2. In addition to eEF1Balpha2 we also considered UBP6 because these two reference genes have been used previously to test Fe deficiency-induced responses in Arabidopsis plants (Wang et al., 2007). All amplification plots were analyzed with Mx3000P software to obtain Ct values. Relative RNA levels were calibrated and normalized (geometric average) with the level of both UBP6 and eEF1Balpha2 mRNA. qPCR data are expressed as fold change (2^–ΔΔCt^) with respect to the control (GMF-exposed plant grown under full nutrient media).

### Statistical Analysis

Pair comparison analysis (*T*-test) and one-way or two-way analysis of variance (ANOVA) with mean comparisons using Tukey’ test were conducted by using SPSS and Past3 software. Data represent means of at least three independent biological replicates. Each replicate was characterized by pooled tissues (root or shoot) from 20 to 30 plants. Different letters above bars indicate statistically significant differences (*p* < 0.05).

## Results

### Reduction of the GMF to NNMF Prompts Fe Deficiency-Induced Responses in Arabidopsis Plants

**FIGURE 1:**
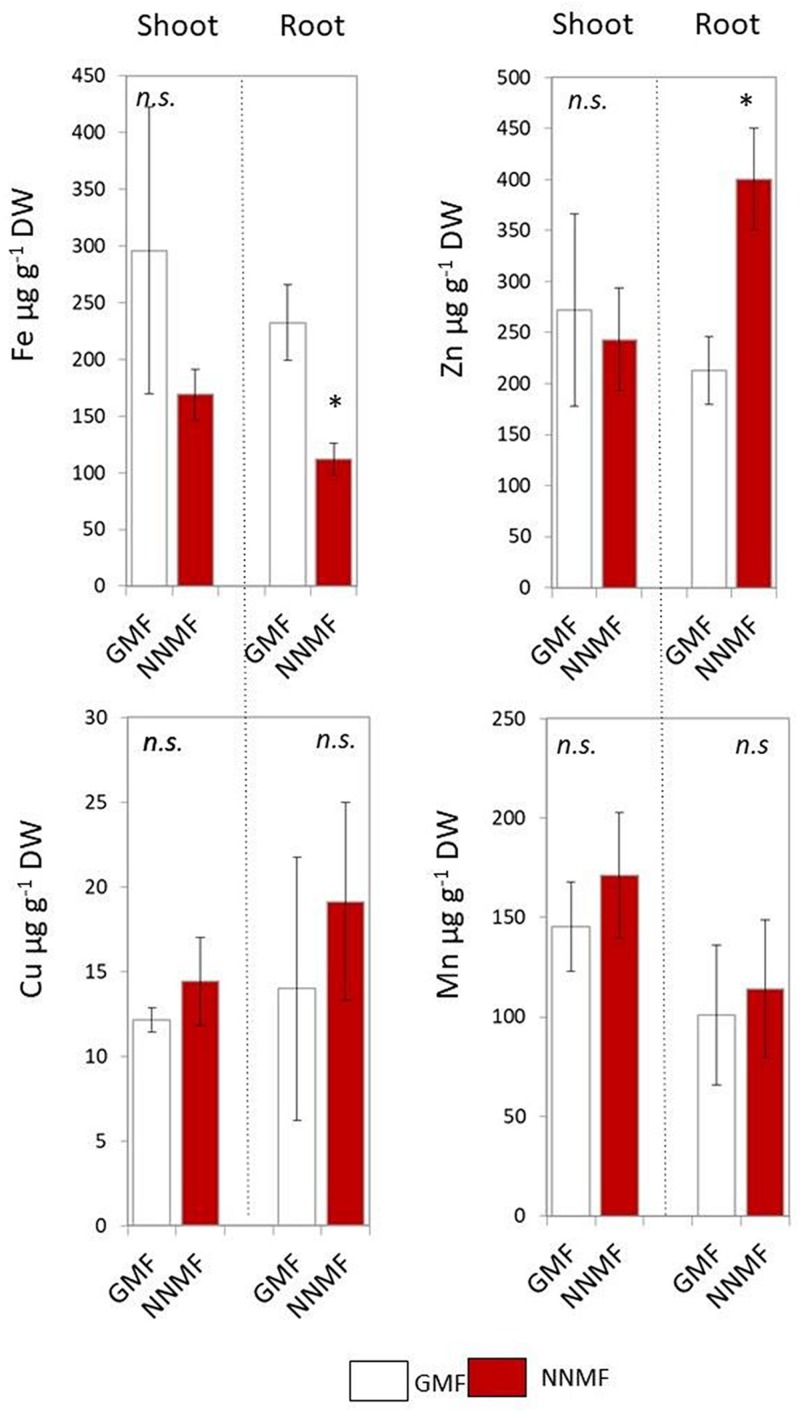
Metal contents (Fe, Zn, Cu, and Mn, expressed as μg/g DW) of shoot and root tissues of *Arabidopsis thaliana* seedlings grown under GMF and NNMF in Fe sufficiency (50 μM FeEDTA) condition. Data are shown as the mean (±SE) from four independent biological replicates (*n* = 4), whereas the asterisk indicates significant (*T* test, *p* < 0.05) differences between NNMF and GMF plants.

Arabidopsis seedlings exposed to NNMF for 96 h displayed an evident variation in metal contents when compared to GMF-exposed plants. In particular, NNMF-treated plant roots showed a significant (*p* < 0.05) decrease in the Fe content and a significant (*p* < 0.05) increase in the Zn content. On the other hand, no significant difference (*p* > 0.05) was observed for both Cu and Mn content. At the shoot level, metals did not display significant differences in their contents under NNMF condition, when compared to GMF control pants (Figure 1).

**FIGURE 2:**
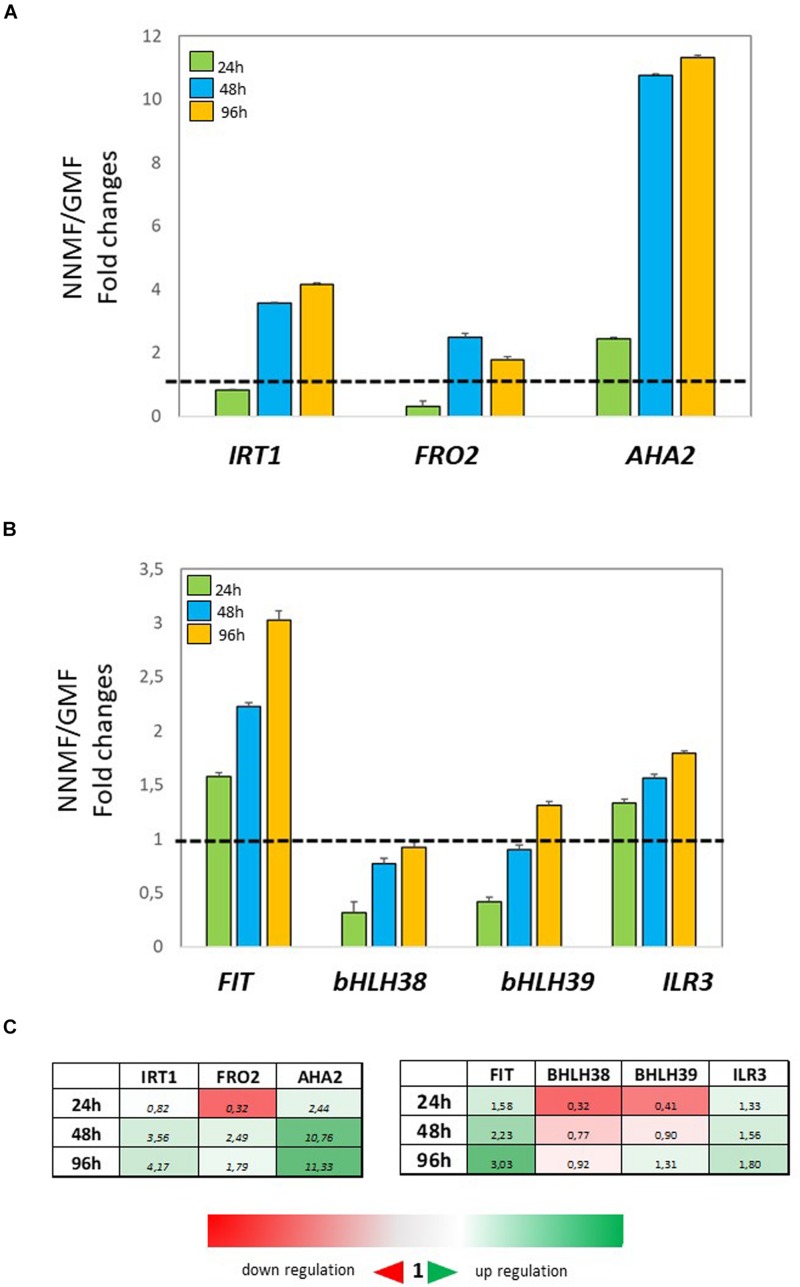
Expression of Fe deficiency-induced genes in roots of *Arabidopsis thaliana* grown under Fe sufficiency condition (Fe 50 μM) and exposed to both GMF and NNMF conditions for 24 h, 48 h, and 96 h. The expression of *FRO2, IRT1, AHA2* genes is reported in **(A)** while the expression of *FIT, bHLH38, bHLH39, ILR3* transcription factors is reported in **(B)**. **(C)** Summary table reporting the qPCR means values. The different color indicates the relative expression levels under the different conditions considered. Data are from three independent biological replicates (*n* = 3). The data were normalized to two internal controls, *eEF1Balpha2* and *UBP6* genes. The relative expression ratios were expressed as NNMF/GMF **(A)** and as –Fe/+Fe (B) fold change (2^–ΔΔCt^) with respect to plants grown in GMF conditions in the presence of Fe [+Fe, 50 μM Fe(III)-EDTA] at given timing points (black dotted line).

In order to investigate the effect of NNMF condition on root metal content, the expression of some Fe uptake-related genes (*FRO2*, *IRT1*, and *AHA2*) as well as some transcription factors involved in their regulation (*FIT*, *bHLH38*, *bHLH39*, *ILR3*) was investigated in time-course experiments. The expression of *IRT1* displayed more than 3-fold increased 48 h and 96 h after NNMF exposure; *FRO2* expression showed a 2-fold increase mainly after 48 h, and *AHA2* expression displayed a 10-fold increase 48 h and 96 h after NNMF exposure (Figures 2A,C). Concerning the transcription factors considered, only *FIT* expression displayed strong induction (more than 2-fold) in root tissues mainly after 48 h and 96 h of NNMF exposure when compared with GMF condition (Figures 2B,C). On the other hand, a down-regulation (2-fold decrease) of *FRO2*, *BHLH38*, and *BHLH39* occurred in short time exposure of plants to NNMF (Figures 2A–C). In agreement with metal content analysis, such results indicate that plants exposed to NNMF faced Fe deficiency conditions.

### Reducing the GMF to NNMF Rapidly Affects Root Metals Reductase Activity *in vivo*

**FIGURE 3:**
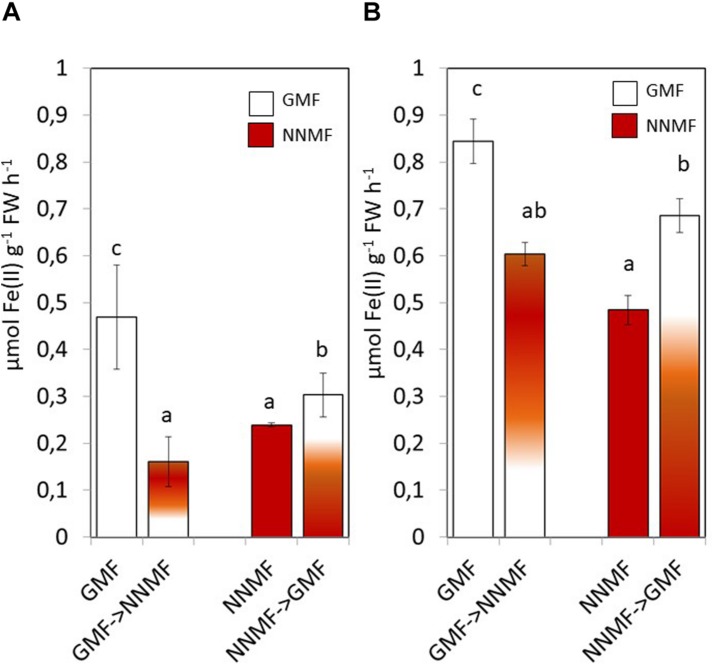
*In vivo* root ferric reductase activity of *Arabidopsis thaliana* grown under Fe sufficiency **(A)** and deficiency **(B)** conditions. Data are shown as the mean (±SE) of six independent biological replicates (*n* = 6). Different letters indicate statistical difference (*p* < 0.05) among samples.

Having assessed that NNMF-exposed plants display modulation of Fe uptake system genes earlier, we monitored the effect of MF variation on the root Fe(III) reductase activity 30 min after transferring GMF-grown plant to NNMF under Fe sufficiency conditions (Figure 3A). We observed that root Fe(III) reductase activity of GMF-grown plants significantly (*p* < 0.05) decreased under 30 min exposure to NNMF compared with plants under GMF conditions. However, the effect was not reversed, and Fe(III) reductase activity still decreased after transferring 30 min NNMF-grown plants to GMF conditions (Figure 3A). We also assayed Fe(III) reductase activity in plants grown under Fe deficiency, which showed a similar trend as observed under Fe sufficiency only when plants were transferred from GMF to NNMF conditions (Figure 3B). However, when plants were transferred from NNMF to GMF conditions, we observed an increase of Fe(III) reductase activity (Figure 3B). Overall, our results indicate that root Fe(III) reductase activity is affected by the GMF. Such variation is attributable specifically to the impact of MF on the enzymatic activity, since the chemical Fe reduction performed by using (i) DTT as reducing agent, (ii) FeEDTA as Fe^3+^ source and (iii) ferroZine as Fe^2+^ chelating compound, did not significantly (*p* > 0.05) change by transferring reaction tubes from GMF (0,146 ± 0.1 nmol Fe^2+^ ml^–1^) to NNMF (0,18 ± 0.12 nmol Fe^2+^ ml^–1^).

**FIGURE 4:**
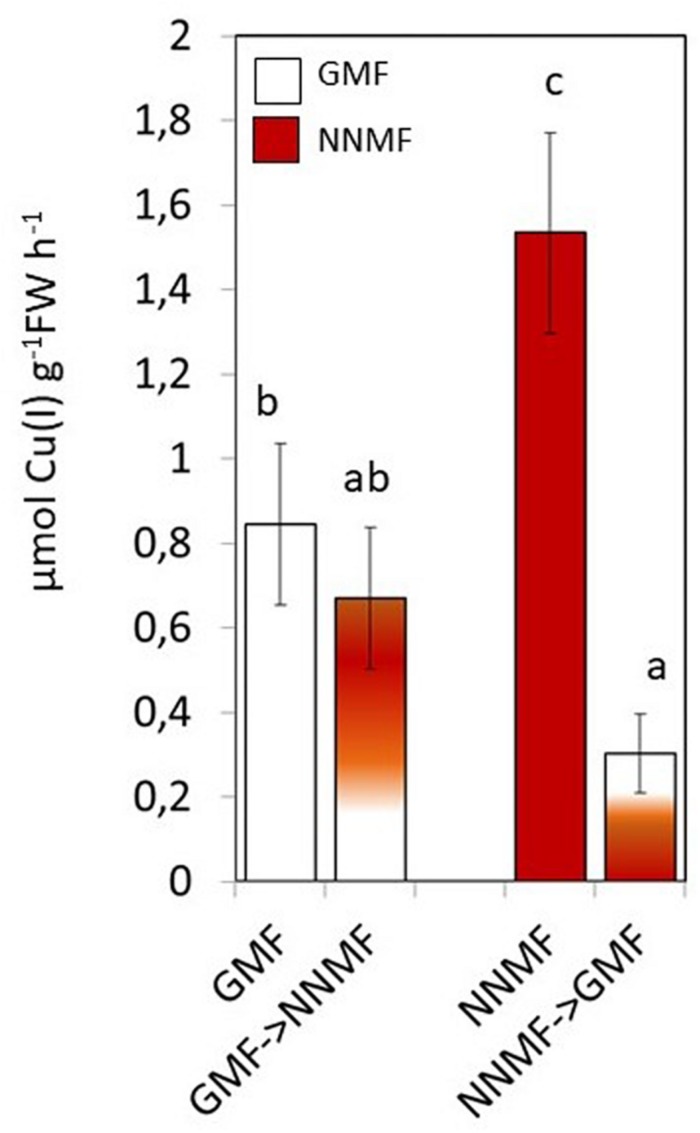
*In vivo* root cupric- reductase activity of Arabidopsis thaliana plants grown under complete nutrients conditions. Data are shown as the mean (±SE) of six independent biological replicates (*n* = 6). Different indicate statistical difference mean (*p* < 0.05) among samples.

Along with Fe, other metals also, such as Cu, require reduction before being taken up by plants. NNMF-exposed plants displayed increased Cu(II) reductase activity when compare to GMF-exposed plants; however, in Cu sufficiency conditions, such activity was not affected by transferring plants from GMF to NNMF. On the other hand, Cu(II) reduction activity was significantly (*p* < 0.05) affected after transferring plants from NNMF to GMF (Figure 4). Similar results were observed on plants grown under Cu deficiency conditions (data not shown). Such results indicate that, although in a different manner if compared to Fe, Cu uptake is also affected by the variation in MF intensity.

**FIGURE 5:**
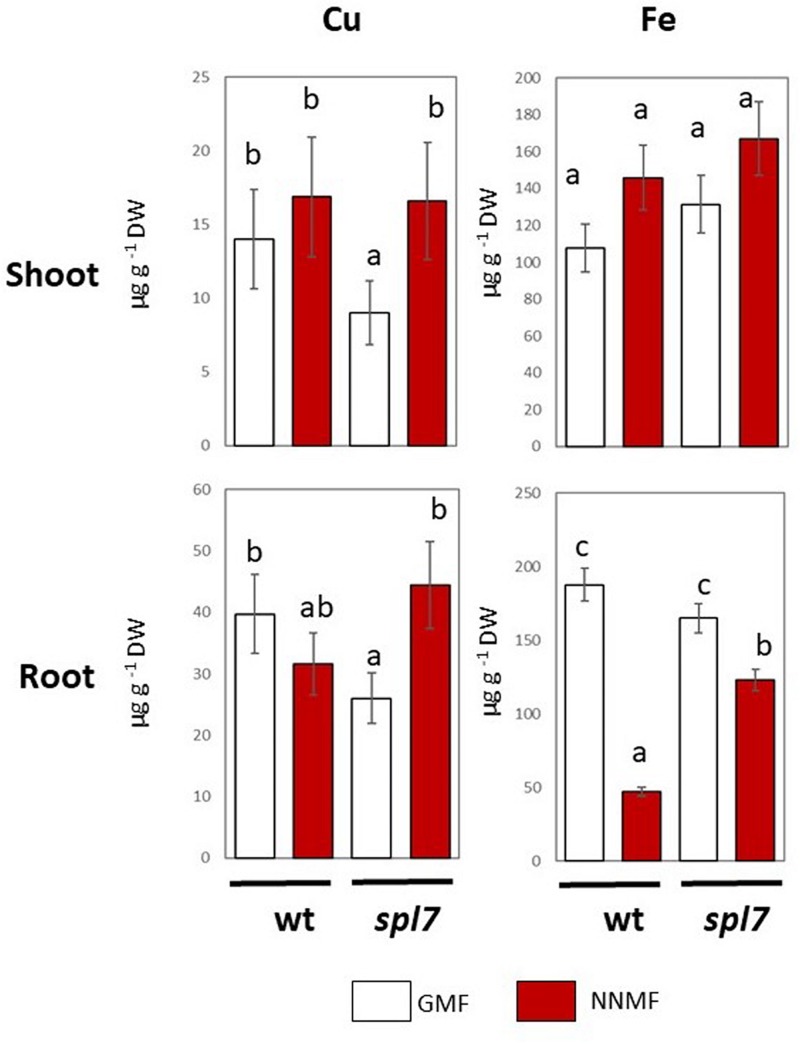
Cu and Fe content in shoot (upper panel) and root (lower panels) of wild type and *spl7 Arabidopsis thaliana* lines. Data are expressed as the mean (±SE) of three independent biological replicates (*n* = 3). Different letters indicate statistical difference (*p* < 0.05) among samples.

In plants, Cu uptake occurs both with a high affinity and a low affinity system (Bernal et al., 2012). The high affinity is a reductive-based mechanism regulated by the SPL7 transcription factor (Bernal et al., 2012). In order to provide further evidence on the effect of the GMF on plant Cu uptake, Arabidopsis *spl7* mutants were grown under either GMF or NNMF. As expected, in both roots and shoots, the Cu content significantly (*p* < 0.05) decreased in the *spl7* mutant, with respect to WT lines under GMF condition (Figure 5). Though, the Cu content did not show significant (*p* < 0.05) variations in the *spl7* mutant, with respect to WT when grown under NNMF. However, under NNMF, Fe content increased in root of *spl7* mutant plants compared with WT plants (Figure 5).

### The GMF Differentially Affects Fe Availability and Plant Growth

**FIGURE 6:**
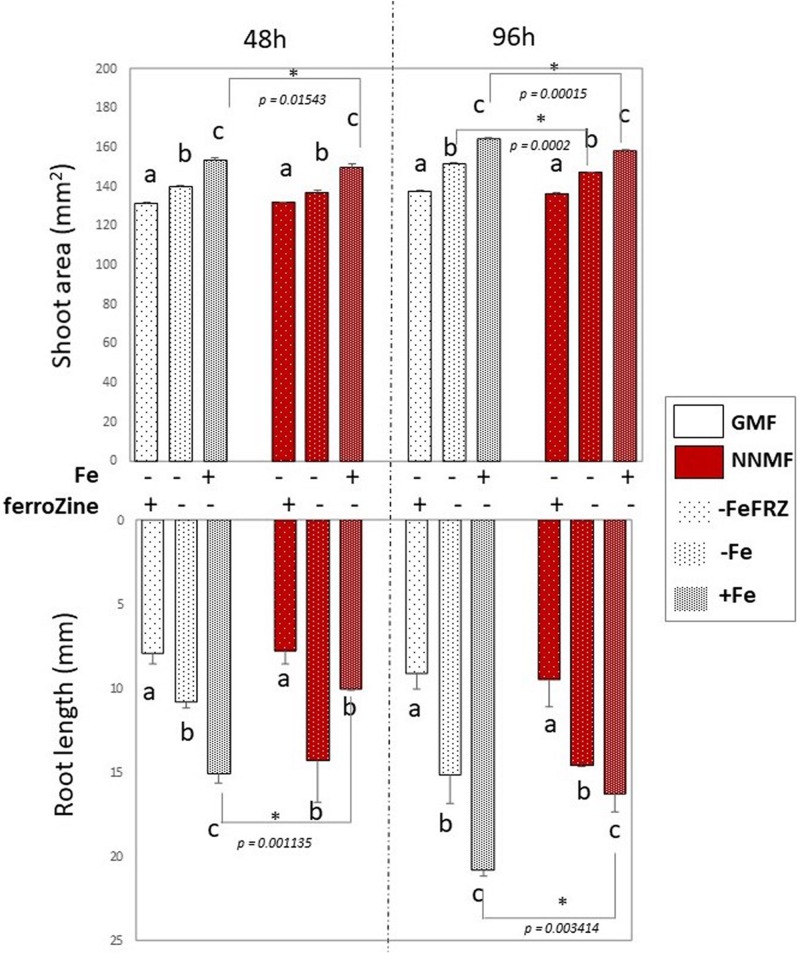
Morphometric measurements (root length and shoot area) of *Arabidopsis thaliana* seedlings grown in the presence [50 μM Fe(III)-EDTA (+Fe)] and in the absence [0 μM Fe (III)-EDTA, (–Fe) and 0 μM Fe (III)-EDTA + 300 μM ferroZine, –Fe+FRZ] of Fe. Root length and shoot area measurement were performed 48 h and 96 h after transferring seedlings to NNMF conditions. Mean value (±SE) are from three independent biological replicates (*n* = 3) and different letters indicate statistical difference (*p* < 0.05) among treatments (+Fe–Frz; –Fe–Frz; –Fe+Frz) within MF groups (GMF and NNMF). Asterisks indicates significant differences between GMF and NNMF plants grown under the same Fe media concentration treatment.

Considering the activation of Fe deficiency-induced genes occurring in NNMF-treated plants, the effect of Fe deficiency on plants exposed to either NNMF or GMF conditions was tested without the addition of Fe in the media as well as by adding ferroZine (which is known to be a specific Fe^2+^ chelator; Gibbs, 1976) to Fe-free media. Growth parameters (shoot area and root length) were determined on plant seedlings exposed for 48 h and 96 h to both GMF and NNMF conditions. As expected, under GMF condition, Fe deficiency affected both shoot area and root length (Figure 6). Such results were also observed in plants grown under NNMF. Two-way ANOVA revealed a significant (*p* < 0.05) interaction between Fe availability and the MF intensity, indicating that the Fe availability of external media affects the ability of plants to perceive variations in MF intensity. In particular, the impairment of plant growth under NNMF was observable only under Fe sufficiency conditions, while low Fe availability was found to limit such effect. Indeed, no difference in root length between GMF and NNMF conditions was observed under both Fe deficiency (−Fe, 0 μM Fe) and ferroZine (FeFRZ, 0 μM Fe + 300 μM ferroZine) treatments (Figure 6). However, a decrease in the shoot area was detected in −Fe plants exposed to NNMF for 96 h when compared with plant exposed to GMF conditions (Figure 6). In plants growing in the presence of ferroZine treatment, we found no significant (*p* > 0.05) differences between NNMF and GMF.

Overall, such results indicated that reduction of the GMF to NNMF affects plant growth mainly in the presence of Fe, highlighting the link between such element and the effect of the GMF.

### Reduction of the GMF to NNMF Modulates Fe Uptake Genes Under Fe Deficiency

**FIGURE 7:**
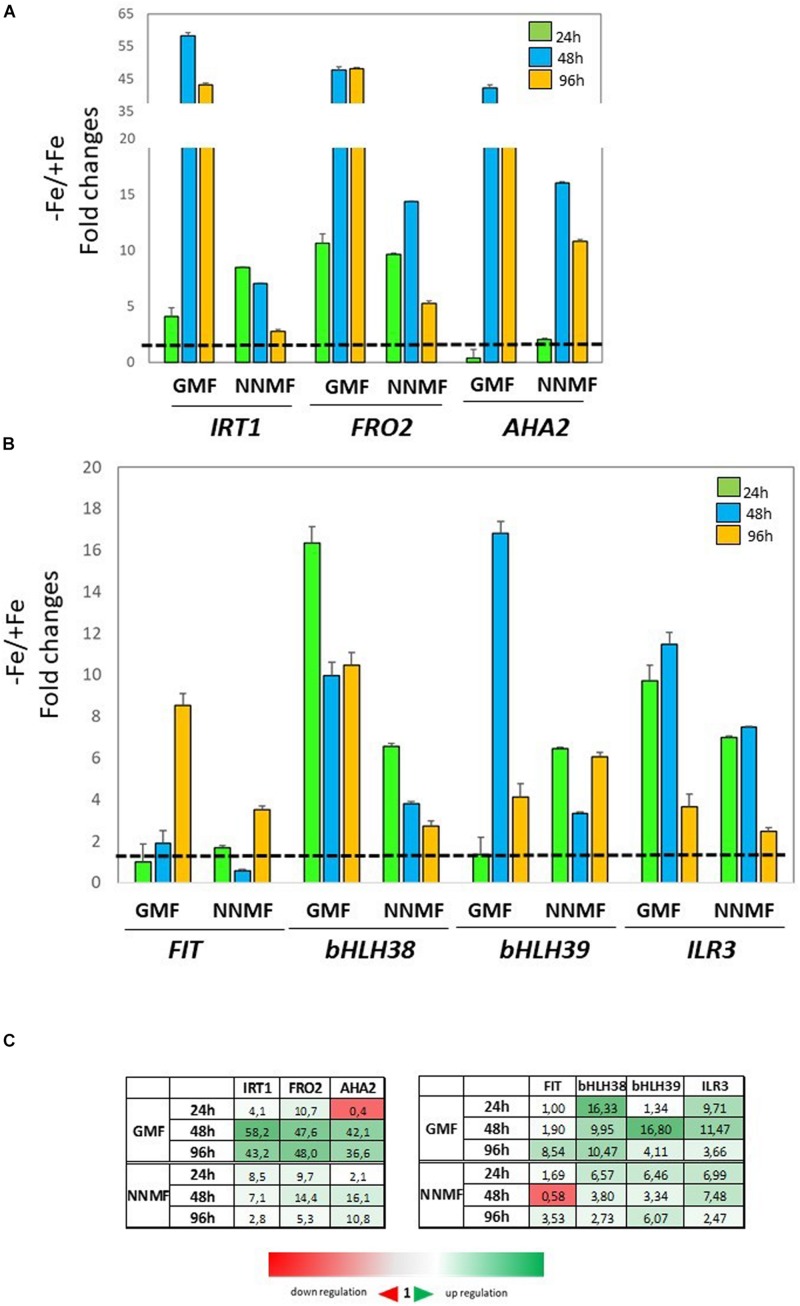
Expression of Fe deficiency-induced genes in Arabidopsis thaliana grown under Fe deficiency condition [0 μM Fe(III)-EDTA, –Fe]. Plants were exposed for 24 h, 48 h, and 96 h both to GMF and NNMF conditions. Expression of *FRO2, IRT1, AHA2* genes is reported in the upper panel **(A)** while the expression of *FIT, bHLH38, bHLH39, ILR3* genes is reported in the middle panel **(B)**. **(C)** Summary table reporting the qPCR means values. The different color indicates the relative expression levels under the different conditions considered. Data are from three independent biological replicates (*n* = 3). The data were normalized to two internal controls, *eEF1Balpha2* and *UBP6* genes. The relative expression ratios were expressed as NNMF/GMF **(A)** and as –Fe/+Fe (B) fold change (2^–ΔΔCt^) with respect to plants grown in GMF conditions in the presence of Fe [+Fe, 50 μM Fe(III)-EDTA] at given timing points (black dotted line).

The expression of *FIT*, *bHLH38*, *bHLH39*, *ILR3*, *FRO2*, *IRT1*, and *AHA2* genes was monitored in Fe-deficient plants exposed to either GMF or NNMF. As expected, under GMF growing condition, genes involved in the plant Fe uptake systems were strongly upregulated under −Fe mainly after 48 and 96 h: *IRT1*, *FRO2*, and *AHA2* expression showed more than 35-fold increase at 48 and 96 h of Fe deficiency (Figures 7A,C). Accordingly, the expression of *bHLH38*, *bHLH39*, and *ILR3* transcription factors displayed al least a 10-fold increase mainly after 48 h of Fe deficiency in GMF condition, while the expression of *FIT* increased by about 8-fold after 96 h of Fe deficiency (Figures 7A,C). Such genes were also induced in plants exposed to NNMF, but to a lower extent compared with Fe-deficient plants exposed to GMF condition (Figure 7). Under NNMF condition, the expression of *IRT1* displayed 8-fold and 7-fold increase after 24 and 48 h of Fe deficiency, respectively, while the expression of *AHA2* displayed a 16-fold and 10-fold increased after 48 and 96 h. Furthermore, the induction of *IRT1* and *FRO2*, decreased from 24 to 96 h of NNMF exposure (Figures 7B,C). Accordingly, also *FIT*, *bHLH38*, and *bHLH39* expressions were induced to a lower extent under Fe deficiency of NNMF-exposed compared with GMF-exposed plants (Figures 7B,C).

Moreover, under NNMF condition, ferroZine-treated plants (-FeFRZ) showed a decrease in Fe content compared with GMF conditions (Supplementary Figure S2) and the expression of Fe uptake genes was differentially affected compared with their expression in root of plants grown under −Fe condition. Yet, the inhibiting effect of low Fe availability on Fe uptake genes in NNMF-exposed plants was less evident in −FeFRZ plants when compared with −Fe treatment (Supplementary Figure S1).

**FIGURE 8:**
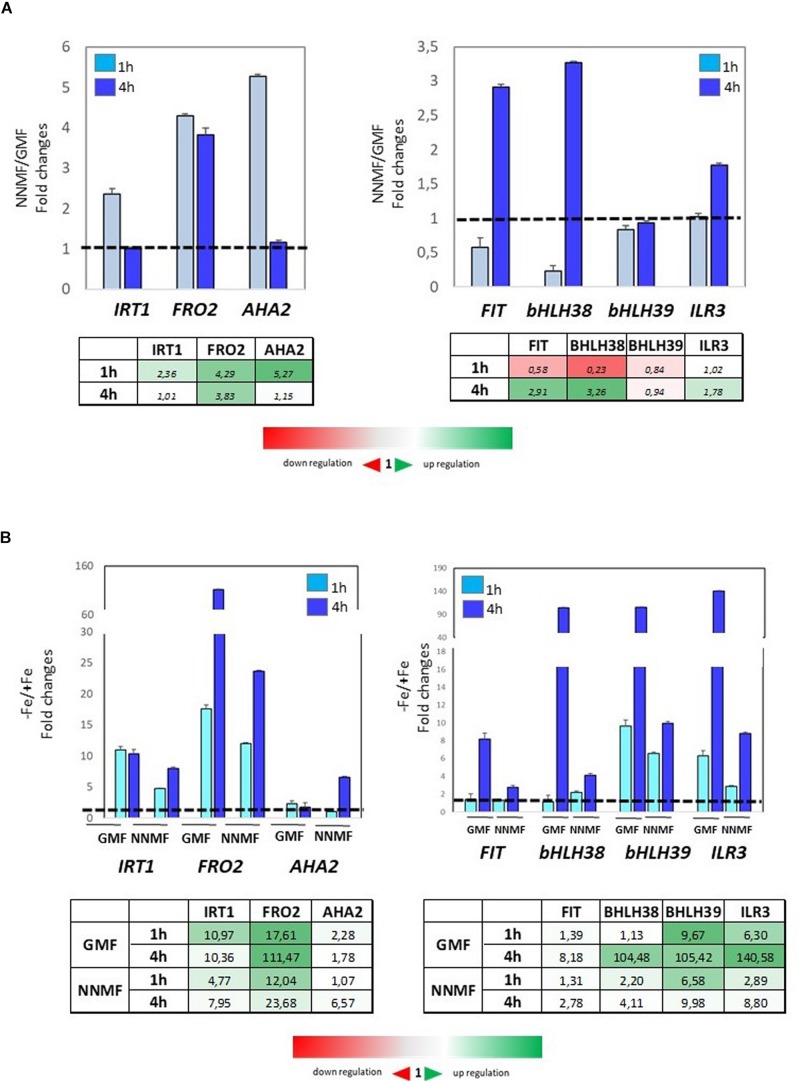
Expression of Fe deficiency-induced genes (*FIT, bHLH38, bHLH39, ILR3, FRO2, IRT1, AHA2*) in *Arabidopsis thaliana* grown both in the presence **(A)** and in the absence **(B)** of Fe and exposed for 1 h and 4 h to both GMF and NNMF conditions. The relative expression ratios were expressed as NNMF/GMF **(A)** and as –Fe/+Fe **(B)** fold change (2^–ΔΔCt^) with respect to plants grown in GMF conditions in the presence of Fe [+Fe, 50 μM Fe(III)-EDTA] at given timing points (black dotted line). Data are from three independent biological replicates (*n* = 3).

To investigate early effects of GMF intensity variation on Fe homeostasis, the expression of Fe uptake genes was monitored at both 1 h and 4 h after transferring plants from GMF to NNMF conditions. Under Fe sufficiency, the expression of *IRT1, FRO2*, and *AHA2* displayed a 2-fold, 4-fold and 5-fold increase, respectively, already 1 h after of NNMF exposure (Figure 8A, right panel). On the other hand, the expression of *FIT* and *bHLH38* was downregulated 1 h after NNMF exposure and, along with *ILR3*, their upregulation occurred 4 h after NNMF exposure (Figure 8A, left panel). Under Fe deficiency, plant exposed for 1 h and 4 h to NNMF displayed a lower induction of *IRT1* and *FRO2* genes when compared with GMF exposed plants, while *AHA2* expression increased about 5-fold 4 h after NNMF exposure respect to GMF exposed plants (Figure 8B, left panel). Accordingly, the expression of FIT, bHLH38, bHLH39, and ILR3 increase at lower extent under Fe deficiency in NNMF-exposed plants compared with GMF-exposed plants (Figure 8B, right panel).

**FIGURE 9:**
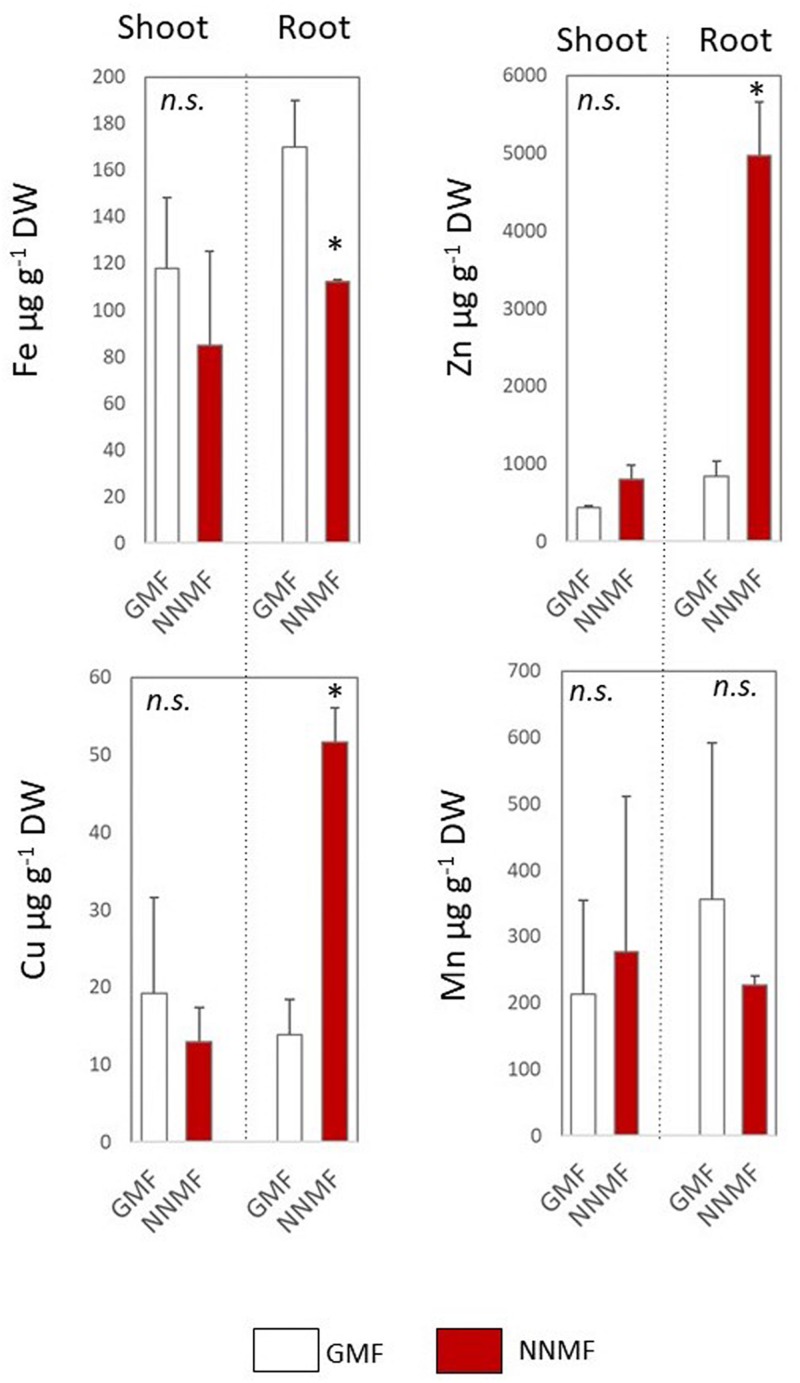
Metal contents (Fe, Zn, Cu, and Mn, expressed as μg/gDW) of shoot and root tissues of Arabidopsis seedlings grown under Fe deficiency (0 μM Fe-EDTA) and exposed for 96 h to both GMF and NNMF Data display mean (±SE) from three independent biological replicates (*n* = 3). Asterisk indicates significant (*p* < 0.05) differences between NNMF and GMF plants.

Under Fe deficiency, *Arabidopsis* seedling displayed a differential impairment of metal content 96 h after exposing them to NNMF, when compared to GMF condition (Figure 9). Along with a decrease in the Fe content, a strong accumulation of both Cu and Zn in root tissues of NNMF-exposed plants was observed, while a no significant (*p* > 0.05) change in Mn content was detected (Figure 9).

## Discussion

The aim of this work was to evaluate the role of the GMF on plant metal homeostasis by investigating the effect of the reduction of the GMF to NNMF condition on the metal uptake, enzyme activity and genes expression in Arabidopsis seedlings. Our results show that NNMF-exposed plants display a low Fe content along with an accumulation of Zn in roots, mirroring the effect of a typical plant Fe deficiency condition, as previously observed (Tomasi et al., 2014; Vigani et al., 2018). Accordingly, NNMF-exposed plants strongly upregulated Fe uptake genes, supporting the need of plants to increase their Fe content. These results suggest that the regulation of Fe uptake genes requires a GMF.

Magnetic fields variations have been found to impair plant redox reaction rates (Bozic et al., 2008; Bertea et al., 2015). Because Fe is involved in several redox reactions in plant metabolisms (Vigani and Murgia, 2018), the reduced Fe uptake in NNMF-exposed plants shown here implies a possible role of the GMF in Fe homeostasis. Moreover, Fe uptake depends on redox reactions carried out by the root ferric reductase activity. By transferring plants from GMF to NNMF, root Fe reductase activity decreased, indicating that the ability of the plant to acquire Fe from the external medium is GMF-dependent. Such an effect would determine a lower plant Fe content, resulting in the upregulation of the Fe uptake systems. Other than Fe, a reduction-based mechanism is also required for the uptake of other metals, like Cu. We observed that transferring plant from NNMF to GMF impaired the root Cu reduction activity. The unaffected content of Cu in Arabidopsis seedlings under NNMF might be linked to the fact that Cu is acquired by two different systems, whereas in Arabidopsis Fe reductase activity is an obligate step to acquire Fe from the soil (Bernal et al., 2012). Copper uptake depends on both a high-affinity system (reductive-based mechanism) and a low-affinity system (not reductive-based mechanism) (Bernal et al., 2012). The response of the Arabidopsis *spl7*-mutant to NNMF condition offered more information on the Cu uptake. SPL7 is a regulator of Cu^2+^ reduction-based uptake in plants as well as a repressor of Fe uptake, mainly under Cu deficiency (Bernal et al., 2012).

Moreover, *bHLH38* and *bHLH39*, along with *FRO2* and *IRT1* are among the SPL7-responsive genes identified in Arabidopsis (Ramamurthy et al., 2018). The observation that both Fe and Cu content increased in roots of the *spl7* mutant under NNMF suggests that the GMF affects the Fe-Cu molecular crosstalk in plants. This hypothesis is also supported by the fact that under full nutrient condition, the reduction of the GMF to NNMF affected the Fe reductase activity and decreased the Fe uptake efficiency of plants by leading to Fe deficiency.

Under Fe deficiency conditions, NNMF impairs Fe uptake systems by lowering the induction of gene expression at a lower extent when compared to GMF exposed plants. We also observed that under NNMF, plants displayed a higher accumulation of metals like Zn and Cu in Fe-deficient roots. It has been recently demonstrated that the excess of some metals (Zn, Co, Cd, and Ni) induce Fe deficiency responses in Arabidopsis plants (Lešková et al., 2017). Therefore, such metals interfere with Fe homeostasis. Particularly, it has been observed that excess of Zn induces plant Fe deficiency (Vigani et al., 2018). IRT1 drives the uptake of both Fe and non-iron metals (i.e., Mn, Zn, Co) and, under Fe deficiency, accumulation of such metals occurs in plants. The absence of IRT1 protein in Arabidopsis has been observed under Zn excess (Connolly et al., 2002) and IRT1 protein levels are regulated post-translationally through ubiquitin-mediated proteasomal degradation (Kerkeb et al., 2008; Barberon et al., 2011; Dubeaux et al., 2018). Moreover, intracellular Zn accumulation mimics Fe deficiency and inhibits plant cysteine oxidase (PCO), which is considered the oxygen sensors in plants (Dalle Carbonare et al., 2019). Hence, we cannot exclude that the alteration of Fe deficiency-induced genes expression in plants exposed to NNMF might be linked to such unbalance of metal uptake occurring under this condition, and work is in progress in order to test this hypothesis. Considering that metals differ from each other depending on their magnetic properties, by devising them into ferromagnetic, paramagnetic and diamagnetic elements, our results suggest that the GMF might be an important factor for mineral nutrients acquisition in plants.

Unbalanced metal content affects plant growth and development by affecting circadian clock regulation (Haydon et al., 2015). Recent evidence demonstrated that the interaction between nutritional status and the circadian clock occurs in plants (Haydon et al., 2015). Particularly, the regulation of oscillator genes is affected by Cu homeostasis impairment (Andrés-Colás et al., 2010; Peñarrubia et al., 2010). On the other hand, Cu homeostasis is strictly connected with other nutrients like Fe in plants (Bernal et al., 2012; Carrió-Seguí et al., 2019) and altered circadian rhythms impact on Fe homeostasis. Optimal Fe level in plants is achieved by the modulation of TIME FOR COFFEE (TIC), a circadian clock regulators modulating the expression of the ferritin gene *AtFer1* (Duc et al., 2009). Furthermore, the impairment of chloroplast development caused by Fe deficiency may play a role in the modulation of circadian period length for plant growth and development (Salomé et al., 2013). Since, it has been recently demonstrated that NNMF modulates the expression amplitude of some clock genes in Arabidopsis plants (Ikeda et al., 2013; Agliassa and Maffei, 2019), our findings allow us to speculate that the impairment of metal homeostasis might be associated to the circadian clock alteration observed under NNMF conditions (Agliassa et al., 2018a; Agliassa and Maffei, 2019).

## Conclusion

Overall this work, provided evidence that (i) the GMF is a contributing factor for efficient iron uptake in plants and hence for optimal plant growth; (ii) low Fe availability differentially impacts on metals contents under different MF intensity conditions, and (iii) reduction of the GMF to NNMF affects metal reductase activity of roots. Recently, it has been observed that plants modulate the ions content in roots a few minutes after exposure to NNMF conditions (Narayana et al., 2018). Such rapid responses of plants to NNMF suggested that transport activity might depend on magnetoreception systems which is not related to gene expression, although channel-related gene expression is affected by NNMF conditions. Accordingly, our findings suggest that the mechanism of plant magnetoreception might involve and/or affect the nutrients uptake processes. However, it is important to consider that variations in nutrients content impair the whole metabolism suggesting that several biochemical processes might be involved in plant responses to altered GMF intensity.

The importance of such findings is also associated to the variability of the GMF with latitude as well as global GMF inversions during life evolution (Maffei, 2014). The importance of the GMF on metals (e.g., Fe) uptake might be also linked to the evolution of Strategy I (Fe reductive-mechanism) and II (non-reductive mechanism) mechanisms in plants (Maschner and Römheld, 1994).

Considering the importance of MF variations in different environments and in future space exploration where plants might experience MF conditions different from the GMF, our results stress the importance to deepen the investigation on the effect of MF on plant nutrients homeostasis in order to understand how magnetoreception occurs in plants and in turn how nutrients availability changes depend on GMF fluctuating values.

## Data Availability Statement

The raw data supporting the conclusions of this article will be made available by the authors, without under reservation, to any qualified researcher.

## Author Contributions

GV and MM conceived and designed the study. MI and GV performed the experiments. MI participate in drafting the manuscript. GV wrote the manuscript. MM revised critically the manuscript. All the authors approved the final version of the article.

## Conflict of Interest

The authors declare that the research was conducted in the absence of any commercial or financial relationships that could be construed as a potential conflict of interest.

## Supplementary Material

FIGURE S1 Effect of NNMF and Fe availability condition on the growth and on the Fe content of Arabidopsis seedlings.

FIGURE S2 Time-course expression of Fe deficiency-induced genes (*FIT, bHLH38, bHLH39, ILR3, FRO2, IRT1, AHA2*) in Arabidopsis thaliana grown under Fe absence condition (0 μM Fe(III)-EDTA + 300 μM ferroZine) and exposed for 24 h, 48 h, and 96 h both to GMF and NNMF conditions. The data were normalized to two internal controls, *eEF1Balpha2* and *UBP6* genes. The relative expression ratios were expressed as NNMF/GMF **(A)** and as –FeFrz/+Fe **(B)** fold change (2^–ΔΔCt^) with respect to plants grown in GMF conditions in the presence of Fe (+Fe, 50 μM Fe(III)-EDTA) at given timing points (black dotted line).

TABLE S1 Composition of growth media.

TABLE S2 List of primers used.
