# Supplementary figures and images for: The Geomagnetic Field Is a Contributing Factor for an Efficient Iron Uptake in Arabidopsis thaliana
Source: Front Plant Sci. 2020 Apr 21;11:325. doi: 10.3389/fpls.2020.00325 (PMC7186349; doi:10.3389/fpls.2020.00325)

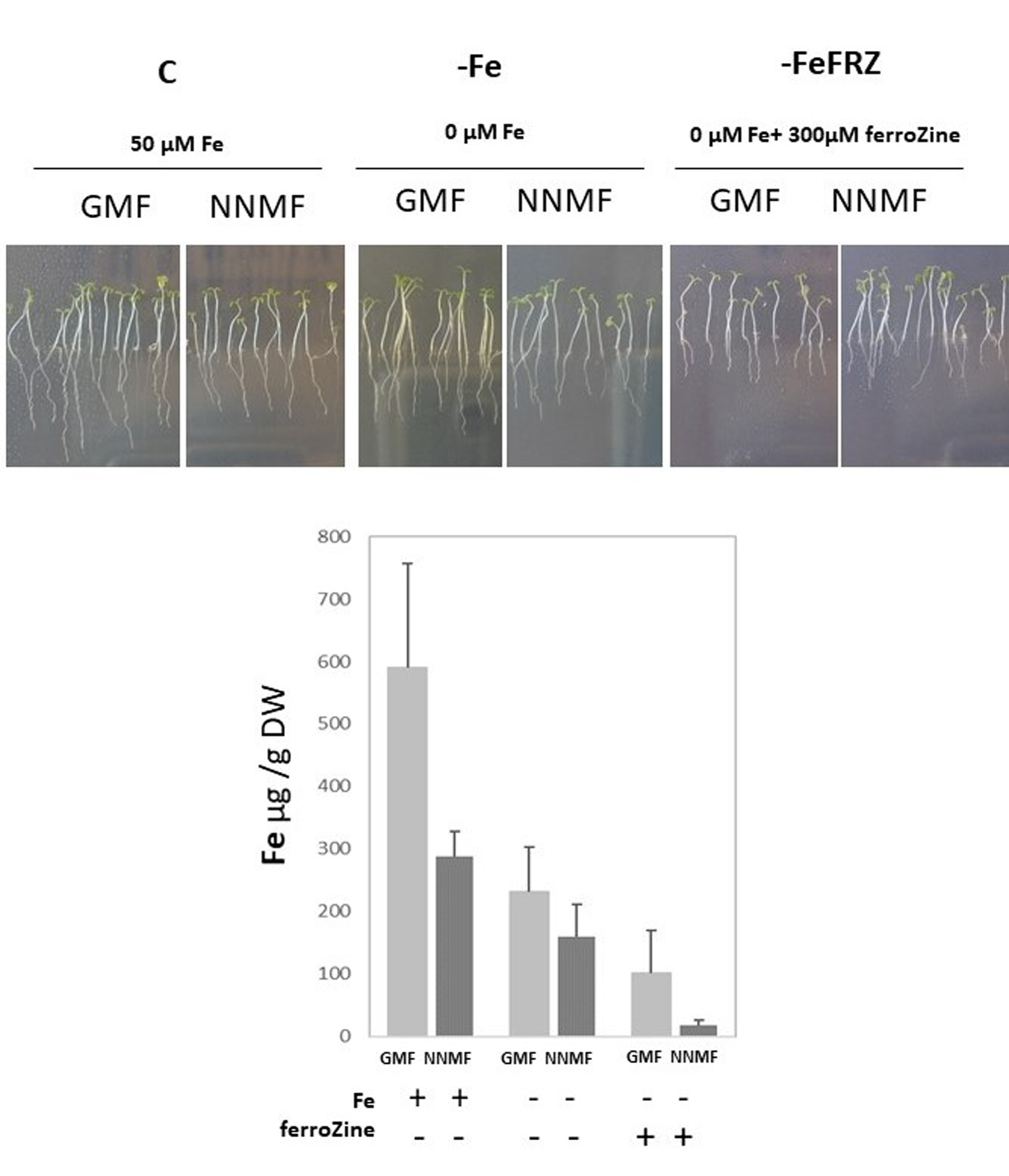

Supplement: FIGURE S1 — Effect of NNMF and Fe availability condition on the growth and on the Fe content of Arabidopsis seedlings. [file Image_1.TIF]

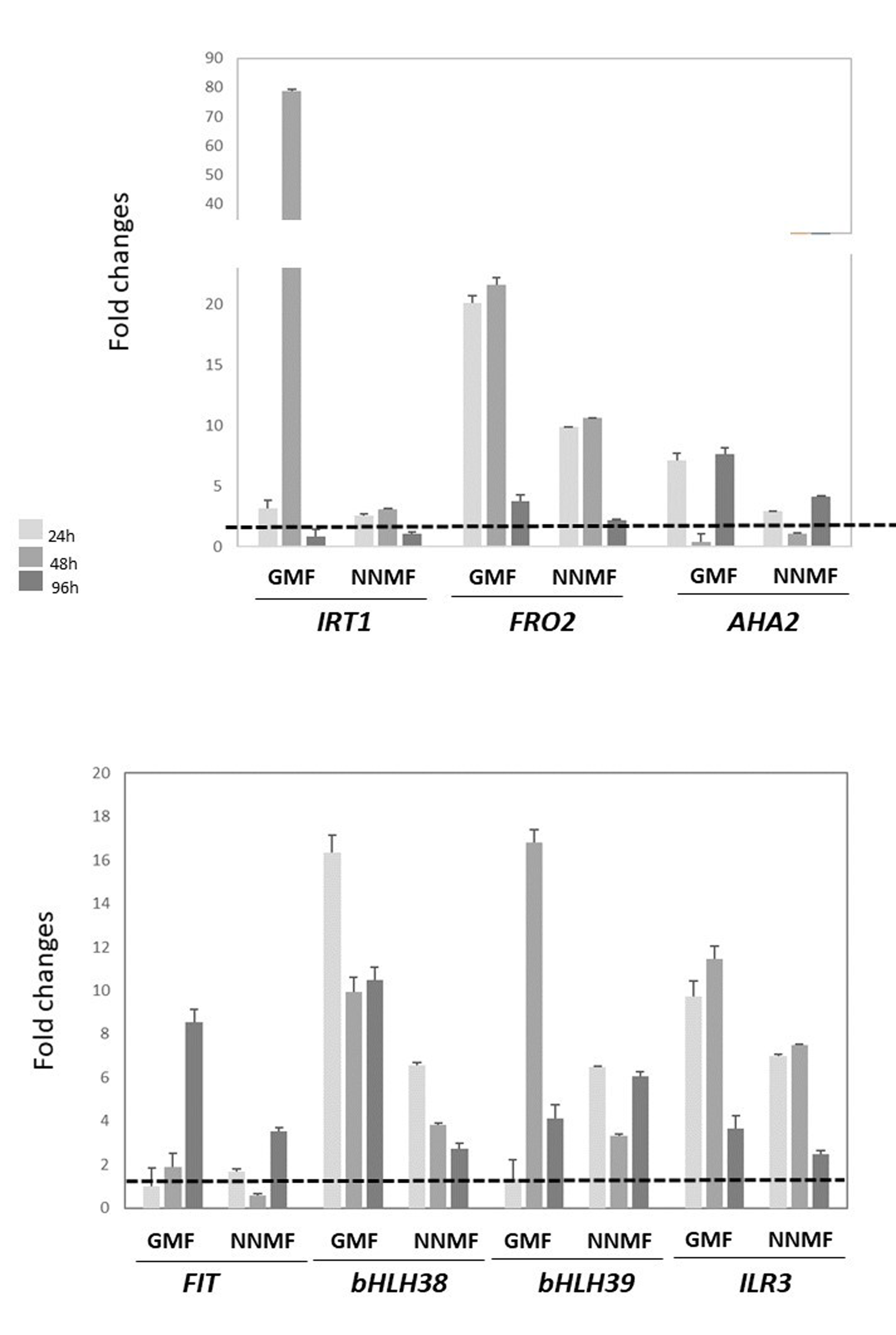

Supplement: FIGURE S2 — Time-course expression of Fe deficiency-induced genes (FIT, bHLH38, bHLH39, ILR3, FRO2, IRT1, AHA2) in Arabidopsis thaliana grown under Fe absence condition (0 μM Fe(III)-EDTA + 300 μM ferroZine) and exposed for 24 h, 48 h, and 96 h both to GMF and NNMF conditions. The data were normalized to two internal controls, eEF1Balpha2 and UBP6 genes. The relative expression ratios were expressed as NNMF/GMF (A) and as –FeFrz/+Fe (B) fold change (2–ΔΔCt) with respect to plants grown in GMF conditions in the presence of Fe (+Fe, 50 μM Fe(III)-EDTA) at given timing points (black dotted line). [file Image_2.TIF]
