# Supplementary material for: The Geomagnetic Field Is a Contributing Factor for an Efficient Iron Uptake in Arabidopsis thaliana
Source: Front Plant Sci. 2020 Apr 21;11:325. doi: 10.3389/fpls.2020.00325 (PMC7186349; doi:10.3389/fpls.2020.00325)
Supplement: TABLE S1 — Composition of growth media. [file Table_1.DOCX]

**Table 1:** Macro and micro nutrient composition

| Name of the nutrients | Concentration µM |
| --- | --- |
| MgSO_4_ 7H_2_O | 750 |
| KH_2_PO_4_ | 625 |
| NH_4_NO_3_ | 1000 |
| KNO_3_ | 9400 |
| CaCl_2_ 2H_2_O | 1500 |
| MES pH5.5 with KOH | 1000 |
| H_3_BO_3_ | 50 |
| KI | 2.5 |
| ZnCl_2_ | 15 |
| NaFeEDTA | 50 |
| CoCl_2_ 6H_2_O | 0.055 |
| CuCl_2_ 2H_2_O | 0.053 |
| MnCl_2_ 4H_2_0 | 50 |
| Na_2_ MoO_4_ 2H_2_O | 0.52 |
| 3-(2-Pyridyl)-5,6-diphenyl-1,2,4-triazine (Ferrozine) | 300 |
