# Supplementary material for: The Geomagnetic Field Is a Contributing Factor for an Efficient Iron Uptake in Arabidopsis thaliana
Source: Front Plant Sci. 2020 Apr 21;11:325. doi: 10.3389/fpls.2020.00325 (PMC7186349; doi:10.3389/fpls.2020.00325)
Supplement: TABLE S2 — List of primers used. [file Table_2.DOCX]

**Table 2:** Primers used in this work

| **Gene Code** | **Gene** | **Forward primer (5’-3’)** | **Reverse primer (5’-3’)** |
| --- | --- | --- | --- |
| **REFERENC GENES** | | | |
| At2g37620 | *ACT1* | TGCACTTCCACATGCTATCC | GAGCTGGTTTTGGCTGTCTC |
| At5g19510 | *eEF1Balpha2* | ACTTGTACCAGTTGGTTATGGG | CTGGATGTACTCGTTGTTAGGC |
| At1g13440 | *GAPC2* | TCAGGAACCCTGAGGACATC | CGTTGACACCAACAACGAAC |
| At1g51710 | *UBP6* | GAAAGTGGATTACCCGCTG | CTCTAAGTTTCTGGCGAGGAG |
| **TARGET GENES** | | | |
| At4g30190 | *AHA2* | AAAGTTGCAGGAGAGGAAGC | GCACGATATCTGAAGCACCA |
| At1g01580 | *FRO2* | TCTCATCAATCCTCGGACCA | TTGTTGGTGTTGTGGTCGAT |
| At4g19690 | *IRT1* | CGGAATAGCGTTAGGGATCG | GCAGCTAGAAGATCCACGAG |
| At2g28160 | *FIT* | GAACATGCTCCTGATGCTCA | ACCCTTTCTCCTCCACTTGT |
| At3g56970 | *BHLH38* | TCAACGGTTTCTGCCACTAG | ACATCCACAAGAACAAACCCA |
| At3g56980 | *BHLH39* | TGTTTCTGTTTCGTCGGAGG | TAATTTTCCTGCGACGGTCA |
| At5g54680 | *ILR3* | GCTGCGAGATGAGAAACAGA | AGTAGGCATCATAGGTGGGG |
| At2g38460 | *IREG1* | TGGGTGGAAATTCTTGGTGG | TGACTCTCCACACATTCCGA |
| At4g22260 | *IMA1* | GCTGCGAGATGAGAAACAGA | AGTAGGCATCATAGGTGGGG |
